# Morphometric and Meristic Characterization of Native Chame Fish (*Dormitator latifrons)* in Ecuador Using Multivariate Analysis

**DOI:** 10.3390/ani10101805

**Published:** 2020-10-04

**Authors:** Ana Gonzalez-Martinez, Mario Lopez, Hebert Mario Molero, Jorge Rodriguez, Martin González, Cecilio Barba, Anton García

**Affiliations:** 1 Department of Animal Production, Faculty of Veterinary Sciences, University of Cordoba, 14071 Córdoba, Spain; agmartinez@uco.es (A.G.-M.); cjbarba@uco.es (C.B.); 2 Agricultural Polytechnic of Manabi, Escuela Superior Politécnica Agropecuaria de Manabi “MFL”, 130250 Calceta, Manabi, Ecuador; mrene782@gmail.com; 3 Research Department, Bolívar State University, Guaranda, 020150 Bolivar, Ecuador; moleroh.molero@gmail.com; 4 Department of Animal Production, Quevedo State Technical University, Av. Quito km. 1 1/2 vía a Santo Domingo de los Tsáchilas. Quevedo, 120501 Los Ríos, Ecuador; jrodriguez@uteq.edu.ec (J.R.); mgonzalez@uteq.edu.ec (M.G.)

**Keywords:** fish species, morphostructure, production system, sex, discriminant analysis

## Abstract

**Simple Summary:**

Ecuador is considered a biodiversity reserve in which 951 native freshwater species of fish are recognized. Most of them have not been characterized and are endemic, inhabiting fragile ecosystems and presenting an endangered situation. In this work, chame was morphostructurally characterized; sex and production systems (cultured and wild) were considered factors of variation. This research represents a first step towards the development of breeding and conservation plans for this native zoogenetic resource.

**Abstract:**

Ecuador, a country exhibiting large developments in fish farming, has a great variety of freshwater native fish. Among these fish is the *Dormitator latifrons* or chame, which has characteristics that make its farming prone to occur at a quite-developed stage. However, morphological characterization is required to establish a conservation program. In this study, 300 chames were captured in the Manabi province (Ecuador) to analyze their morphostructural model and to evaluate the effects of sex and the production system through multivariant techniques. The fish from the farm presented morphological measurements that were statistically (*p* < 0.05) higher than those of wild fish. Males were taller, longer, and wider than females, although the differences were not significant (*p* > 0.05). The percentage of correct adscription was 84%, with larger errors in wild fish. The morphostructural model had a high homogeneity, with 89.95% significant correlations (*p* < 0.05), and wild male and female fish were more homogeneous. The farm fish were larger because of the higher food availability. Moreover, the species exhibited sexual dimorphism, although there were no great differences in the morphometric measurements. This study shows the great biodiversity that naturally exists in Ecuadorian rivers. Therefore, it is of great interest to develop a chame breeding and conservation program.

## 1. Introduction

The *Dormitator latifrons* (Richardson, 1844) or chame is a native American fish with a wide distribution (from California to the Peruvian coast; Figure S1). It grows in tropical and subtropical zones, prefers warm waters with temperatures between 21 and 30 °C and a salinity of up to 14%, has an oxygen tolerance of up to 0.4 ppm, has a pH between 6.4 and 9.4, and has a breeding density of five specimens per m^2^ [1]. Chame is included in the network list of the International Union for Conservation of Nature (IUCN), being part of the 20% of endangered freshwater fish [2,3,4].

Ecuador is rich in ichthyofauna, with 951 species of freshwater fish [4]. Chame lives in freshwater bodies and brackish water of the Ecuadorian coasts, mainly those of the provinces of Manabi, Guayas, and Los Rios [5]. It is a zoogenetic resource that contributes to the conservation of ecosystems and the improvement of territorial competitiveness [6]. This species has the capability to process detritus into other materials that can be used in other trophic levels, with high palatability, robust body, high resistance to physicochemical changes, and low production cost [4]. These characteristics increase the interest in developing its farming from an ecosystem and territorial approach [7].

In Ecuador, small-scale production is predominant on fish farms, with production that ranges from 800 to 1000 tons [8]. The largest production area corresponds to Manabi province, with a production of 4000 ha, which combines 6000 ha of shrimp farming [9]. The production is mainly focused on the growing of juvenile fish; it has a zootechnic characteristic that limits the standardization of reproduction under controlled conditions and the continuous production of seeds [9]. Fish farming in different kinds of water bodies, such as water mirrors, ponds, geomembrane ponds [10], and biofloc systems [11], has been attempted. Farming has even been combined with other activities; for instance, the production of rice–chame [5]. However, the progressive growth of productive activity has a direct effect on the amount of juvenile fish (6’12 cm) that live in a natural way in estuaries, drainage channels, and shrimp pools [12]. Most of them are smallholders that use the water mirrors formed during the rainy season. They use low densities, developing different productive phases from fingerling production to fattening, and the use of balance is limited. Additionally, new technical entrepreneurial actions, using geomembrane pools and oxygenators, are beginning to emerge. They focus their activity on the fattening of fingerlings from other farms, especially shrimp farms. They increase rearing densities to 25 fingerlings per m^2^ and use feed throughout the fattening period. 

The breeders’ census shows a continuous reduction in capture frequency and the quality of the captured animals [9]. Revelo [2] reported a decrease in capture frequency in Los Rios province (only 0.03%). This is an indication of the reduction in the size of the captured animals and a loss of genetic variability [2]. Likewise, this problem is intensified by the exchange of fingerlings among river basins. This has been happening in Latin America since the beginning of the 1960s; aquaculture is one of the major motives of this phenomenon. Apart from this, fish farming has boosted the introduction of foreign species in Ecuador with a high reproductive index, with *Oreochromis spp.* (tilapia) being the most extended case of this.

In fact, there is a growing interest in the preservation of ecosystems and the biodiversity of the ichthyofauna of Ecuador, which is a critical part of the genetic heritage of humanity and a key element to cushioning climate change [7]. Chame is more than a source of protein related to the local rural population, associated with problems coming from food sovereignty, it is an important part of the ecosystem, with a low level of oxygen use and high organic matter [5].

Information about chame is limited, its reproductive biology is unknown, and its process of production is nonstandardized [5]. This information must be discovered before the development of a conservation program [13], which will include the restocking and recovery of ecosystems with native fingerlings of the species [7]. It is also necessary to maintain the way of life of the fishermen and the rural population associated with it. In relation to this situation, on the one hand, the morphological and meristic characterization of chame is needed; on the other hand, this characterization is in an advanced stage compared to other native species [14,15], all of which are interesting to conservationists. 

Therefore, the aim of this study is to conduct the morphometric and meristic characterization of *Dormitator latifrons*, considering sex and the production system as factors. Additionally, morphostructural differentiation amongst groups (males and females from cultured and wild systems) through discriminate analyses was evaluated. 

## 2. Materials and Methods 

### 2.1. Study Sample and Data Collection 

This study was conducted during the rainy season (April to July of 2016) in the province of Manabi (Ecuador), which has a tropical climate with an average temperature of 25.6 ºC, annual precipitation of 838.7 mm, and relative humidity of 78%. The studied fish came from a river (wild) and a farming pool. The wild fish were caught from different locations within their natural geographic distributions in the Simbocal estuary, which is formed by the Carrizal and Chone rivers, from the basins of Chone and Rio Grande, while the farmed fish came from Rancho Viejo farm. Both sites are located in Chone Canton in the province of Manabi (Figures S2 and S3). A total of 300 hundred mature specimens of *Dormitator latifrons* were used: 150 came from direct fishing in the river (78 males and 72 females), and the other 150 came from fish farming (75 males and 75 females). In the river, diurnal catches were made by native fishermen, and the fish were caught using traditional fishing, where the fishnet is swept in the opposite direction to the flow of water. The fish were deposited in a cage attached to the network and were placed in a transition pond under natural conditions to transport them alive. All fish were captured early in the morning using traditional fishing practices following the Konings [16] methodology. On the farm, fish were caught using standard fishing gear. Farm fish were fed three times per day, adjusting the consumption to 1.5% biomass. The cultured fish were fed with different commercial food for prawns, depending on the reproductive stage (Table S1), produced by Agripac S.A., whilst wild fish ate natural food based on phytoplankton and zooplankton. Males and females were differentiated by morphological characteristics; males have a genital papilla with a triangular shape, while in the case of females, this papilla is rounded and presents villi. Moreover, the coloration of each sex is different, being reddish in males and greenish-blue in females. 

In the lab, the fish were kept in two tanks of 500 L each (dissolved oxygen = 6.20 ± 0.0 mg/L, temperature = 20.5 ± 0.2 °C, and pH = 5.6 ± 0.1). Once captured, the fish were stored in water tanks with the purpose of keeping them alive. The fish were maintained without food for 24 h before their death. After that, they were stunned by immersion in a mixture of water and ice at 0.8 ºC for 20 min. After this time and once the death of each fish had been certified, fish were labeled and weighed with an electronic balance with a 0.1 g precision. Then, the morphometric measurement and meristic counts were conducted. The procedure was carried out according to the Canadian Council on Animal Care guide for investigative fish management [17].

### 2.2. Body Measurements

**Figure 1:**
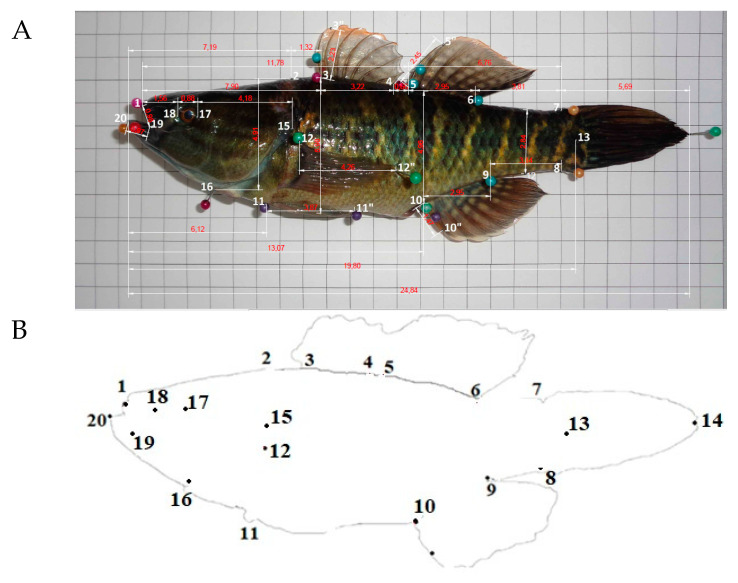
(**A**) The morphometric measurement records for each analyzed organism (source: own elaboration) and (**B**) the location of 20 anatomical landmark points^1^ designed based on the left view of the *Dormitator latrifrons*. 1: most cranial point of the upper premaxilla; 2: nape, most caudal point of head; 3: origin of dorsal_1 fin; 4: ending of dorsal_1 fin; 5: origin of dorsal_2 fin; 6: ending of dorsal_2 fin; 7: dorsal origin of caudal fin; 8: ventral origin of caudal fin; 9: ending of anal fin; 10: origin of anal fin; 11: origin of pelvic fin; 12: origin of pectoral fin; 13: most cranial point of caudal peduncle; 14: most caudal point of caudal peduncle; 15: end of operculum; 16: down of operculum; 17: posterior edge of the eye; 18: anterior edge of the eye; 19: commissure of the mouth; 20: most cranial point of the lower premaxilla.

Morphometric measurements were taken by the same person from the left side of the fish, except for the widths and perimeters, following the conventional method described by Diodatti et al. [18]. Animals were measured using an ichthyometer, graduated digital calipers, and tapes with a 0.01 mm precision. A total of twenty-seven morphometric measurements were obtained, using 20 landmarks and 12 meristic counts (Table 1 and Figure 1), in agreement with the methodology used for native species of Ecuador [14,15]. 

### 2.3. Fulton’s Condition Factor (K)

Fulton’s condition factor (K), which is defined as the wellbeing of fish, was calculated using the equation K = 100 BW/SL^3^, where BW is the total weight (g) and SL is the total length in cm. K is a useful tool for monitoring feeding intensity, age, and growth rates [14]. 

### 2.4. Statistical Analysis

To perform the statistical analysis, the software Statistical 12.0 for Windows was used. Each sample was considered a priori as a discrete group. Before performing the statistical analysis, the Kolmogorov–Smirnov (with the Lilliefors correction) test was applied to verify the normality of the distribution. Most of the variables fit a normal distribution. For those variables that did not show a normal distribution, the Bartlett test was applied to check whether the data had the same variance. 

The morphometric (continuous) and meristic data (discrete) were analyzed separately. The meristic characteristics are independent of the size and do not change during growth [19]; therefore, they were analyzed without further transformation. However, in order to avoid biases due to the size of the morphometric variables, all of them were standardized following the methodology of Elliot et al. [20], which was M_adj_ = M(L_s_/L_o_)^b^, where M is the original value of the morphometric measurement, M_adj_ is the adjusted size of the measurement, L_o_ is the standard length of the fish, and L_s_ is the mean of the standard length of all fish. The parameter b was calculated for each characteristic from the data observed from the regression curve of log M in log L_o_, using all the fish. This method normalized the individuals of a sample with a unique arbitrary size common to every sample, and, at the same time, maintained the individual variation [21]. This adjustment has been applied satisfactorily by several authors [14,15,19]. The efficiency of the transformation was evaluated through the correlation between the transformed variable and the standard length of the fish. Likewise, the transformation of the variables was applied according to Reist [22]; due to the fact that the effect of the size of the fish was not totally removed, the applied equation was M_adj_ = M/SL, where M is the original value of the morphometric measurement, M_adj_ is the adjusted size of the measurement, and SL is the standard length of the fish.

The relationship between morphometric variables was evaluated by Pearson’s correlation coefficients. The morphometric measurements were compared through a multivariate analysis of variance (MANOVA) and the meristic characteristics through the Kruskal–Wallis test, establishing the production system (cultured or wild) and sex (males and females) as the fixed factors, with one degree of freedom for each factor. For the transformed variables, a discriminant analysis was performed, including a graphic representation of the distances of Mahanalobis through clusters, establishing a classification variable with four categories according to the production system (farmed and wild) and sex (males and females). The significance level considered was *p* < 0.05.

## 3. Results and Discussion

### 3.1. Morphometric and Meristic Characteristics

Chame displayed a weight of 173.13 g, which was quite a lot lower than that reported in Ecocostas [12] (1–3 lb; 453.592–1360.78 g), while the total length (24.65 cm) was in the interval reported by those authors (20–30 cm). The large difference in weight could be due to the density of fish stocking and water quality [12]. Table 2 shows the mean values of the morphometric characteristics according to the production system and sex. The cultured fish displayed significantly higher values (*p* < 0.05) in all morphometric measurements, except for the dorsal_2 fin length, anal fin length, and body width 4. The males were taller, longer, and wider than the females, although the differences were only significant (*p* < 0.05) for the head length, dorsal_2 fin length, dorsal_2 fin ray length, and pelvic fin length. The interaction between both effects was only significant for the dorsal_2 fin ray length (*p* < 0.05). The coefficients of variation (CV) of the sample were high (10–20% in most of the analyzed variables), and they were even higher than 30% in some cases (body weight, dorsal_2 fin length, and anal fin ray length).

The farmed fish had a weight and a total length that were lower than those reported by Florencio and Serrano [23] in cultured chame (969 g and 38 cm, respectively), who obtained the largest specimens in the rainy season, which makes food availability a possible factor. 

Several authors have pointed out the effect of origin on the shape of the fish (in *Engraulis* [21] *encrasicolus*, *Pomatomus saltatrix* [19], *Blicca bjoerkna* [24], *Cichlasoma festae* [14], and *Andinoacara rivulatus* [15]). Some similarities were observed in this work, confirming that the specimens from farms were larger, longer, and wider, and had a larger perimeter, with the exception of anal fin length, while not all the variables were statistically different (*p* > 0.05). These differences can be attributed to the availability of food, the environmental conditions, and the kind of habitat [14]. Therefore, one of the main reasons for the weight difference in *Clarias gariepinus* might be related to the artificial food provided [25], similar to what was found in *Dormitator latifrons*, which has already been stated by Wimberger [26].

Cultured fish have higher food availability. Therefore, the competition amongst all specimens is lower and the conditions of the waters facing currents are better for chame’s growth, in comparison to stagnant waters. These are the principal factors that allow good growth among farm fish. However, the season also affects fish growth, having high growth rates while the water level is high (during floods and the early dry season) and decreased growth rates as the water level falls (later in the dry season) [27,28]. 

The age of fish was not considered, although all specimens were adults. However, previous research pointed out that the largest fish were probably 2–3 years old [29]. The high homogeneity in the total and standard length, with coefficients of variation in both measures being lower than 15%, indicates that the age of caught specimens was quite similar.

The genetic origin of the captured fishes and the pressure of fishing in the river could also have affected the results obtained in this work, as pointed out by Ujjania [30] and Ochoa Ubilla [31]. However, the climatology conditions, which are stronger in rivers than on farms, also influence the size of the fish. 

In relation to sex, chame shows visible external sexual dimorphism, which allows researchers to differentiate between males and females [23]. Furthermore, the coloration is different between males and females (reddish in males and greenish-blue in females) [9]. However, whilst the visual difference was clear, it did not result in significant morphostructural differentiation; only four morphometric variables out of twenty-eight exhibited statistical differences (*p* < 0.05) were found between males and females. This result has been found by other authors in analyses conducted on other species where the effect of sex was studied, such as among *Pomatomus saltatrix* [19] and *Andinoacara rivulatus* [15]. Moreover, the differences between males and females with different morphostructures agree with the results obtained in *Clarias gariepinus*, where high phenotypic plasticity was reported inside each sex [25].

The evaluation of both effects—the production system and sex—did not show the existence of significant differences (*p* > 0.05) except for dorsal fin ray length_2. However, the differences in weight and length between the males and females of each production system were higher; the wild and cultured males had a weight and total length of 137.05 g and 23.03 cm and 218.32 and 26.59 cm, respectively. Furthermore, the wild and cultured females had a weight and total length of 135.65 g and 22.65 cm and 201.46 g and 26.33 cm, respectively. These results are in agreement with those reported in *Clarias gariepinus* [32] and *Andinoacara rivulatus* [15]. However, these results are different from those reported in *Clarias gariepinus* [25]. This study identified significant differences (*p* < 0.05) in both the weight and total length, which allowed the authors to claim that the studied sample displayed morphological changes that may have been due to a better adaptation to environmental conditions. The coefficients of variation of the morphometric variables observed in *Dormitator latifrons* may be the result of a differentiated morphostructure, which depends on origin—wild or cultured—as well as sex. 

The mean frequencies of the measured meristic characteristics are shown in Table 3. The number of thorny rays in the two dorsal fins (DFR-1 and DFR-2) displayed values between 5.0 and 6.0 and 8.0 and 10.0, respectively; 98% of the fish had values of 6.0, 8.0, and 7.0, and 96% of them had values of 9.0, 5.0, and 19.0, respectively. 

In terms of the number of cartilages in the dorsal fin (RDF-1 and RDF-2), it fluctuated between 9.0 and 11.0 and 14.0 and 16.0, respectively. A total of 98% of the fish had 5.0, 8.0, and 6.0 cartilages in RDF-1, while 96% of them had 8.0, 5.0, and 9.0 cartilages in RDF-2. In terms of the pectoral fin, the number of thorny rays was between 13.0 and 16.0; 76% of the fish had between 14.5 and 15.0 rays, while the number of cartilages was between 13.0 and 15.0, and 76% of them had between 13.5 and 14.0 cartilages. The pectoral fin showed that all of the studied fish had 5.0 rays and 4.0 cartilages. The number of rays and cartilages of the anal fin oscillated between 10.0 and 12.0 and 9.0 and 11.0, and 78% of the fish had between 10.5 and 11 rays and 9.5 and 10.0 cartilages, respectively. Finally, the tail fin displayed numbers between 13.0 and 16.0 rays and between 12.0 and 15.0 cartilages. Based on these results, 78% of the fish had between 14.0 and 15.0 rays and 14.0 and 15.0 cartilages. The meristic counts showed little differentiation in terms of origin and sex. In relation to the variation coefficient, the meristic characteristics exhibited little variation, with a value lower than 6%.

The meristic variables did not depend on fish size, while the origin and sex exhibited little influence, in concordance with the findings reported by Gonzalez et al. [14] and Caez et al. [15] in *Cichlasoma festae* and *Aequidens rivulatus*, respectively. Conversely, in *Clarias gariepinus,* the differences were significantly higher (*p* < 0.05), although the variations in the meristic characteristics could be small or nil [25]. 

The mean value of the body weight/standard length ratio was 8.84 ± 0.21, with a coefficient of variation of 23.81% (Table 4). The proportions of the head length (HL), corporal perimeter (P1, P2, and P3), body width (LC1, LC2, LC3, and LC4), and body depth (AC1, AC2, and AC3) with respect to the standard length (SL), were 34%, 77–36%, 17–6%, and 33–15%, respectively. The variability was high for the dorsal_2 fin length/standard length, LC4/SL, and AFL/SL ratios, and all the other coefficient ratios had coefficients of variation lower than 15%, including some which were lower than 5% (total length/standard length and preanal length/standard length ratios). The cultured fish displayed higher values for every ratio, with the exception of AFL/SL and P3/SL ratios, while smaller values were obtained for wild females. These differences were statistically significant (*p* < 0.05) for the effect of the production system, while the effect of sex was only significant for five ratios, and the interaction of both effects was only significant for the AC3/SL ratio.

In this work, the BW/SL ratio was significantly higher (*p* < 0.05) in the cultured fish, in agreement with the data obtained for *Cichlasoma festae* [14], but in disagreement with the results reported for *Aequidens rivulatus* [15]. The TL/SL ratio was significantly superior (*p* < 0.05) in males; however, there were no differences between the production systems, which disagree with the data obtained for *Aequidens rivulatus* [15]. Solomon et al. [24] found significant differences (*p* < 0.05) in the HL/SL ratio between wild and cultured *Clarias gariepinus*; instead, in *Dormitator latifrons*, sex affected this ratio, and the same was true in *Aequidens rivulatus* [15]. The head is where larger differences appeared between the wild and cultured samples [33]. The value of the HL/SL ratio and other relations among the morphometric measurements are closely related to the species. For this matter, Gonzalez et al. [14] obtained mean values for the HL/SL ratio that were between 0.31 and 0.45 in *Cichlasoma festae*. In the present work, this ratio was between 0.29 and 0.40.

The confinement of the farm fish affects the fish’s development, mainly due to the lack of stretching of its body, which results in a high value for Fulton’s factor K [33]. This fact is in agreement with the data obtained for *Dormitator latifrons*, while it is in disagreement with those obtained from *Cichlasoma festae* [14] and *Aequidens rivulatus* [15]. However, Turán et al. [19] explained their results in terms of the adaptation process and improvement in the growing process of the fish, which were positively influenced by the supplied conditions in a controlled environment or confinement. 

### 3.2. Relationships Among Morphometric Measurements 

Several positive and significant correlations were observed (*p* < 0.05), and 89.95% of the correlations were significant (*p* < 0.05) (Tables S2 and S3). Taking into account both analyzed factors—the production system and sex—the males and females from cultured fish presented 66.14% and 37.04% significant correlations (*p* < 0.05), respectively, while the wild males and females showed a larger homogeneity of morphostructural shape, with 77.25% and 72.22% significant correlations (*p* < 0.05), respectively. Correspondingly, more than 75.00% of the correlation coefficients were higher than 0.50.

The morphometric relationships among different body parts of the fish can be used to determine possible differences between separated populations of the same species [34]. Solomon et al. [25], in their study on *Clarias gariepinus*, comparing wild specimens with farmed ones, found differences in the correlations which may be strongly related to feeding patterns, morphometric plasticity, environmental stressors, and genetic variability.

The high correlations among the morphometric measurements are in agreement with the results obtained in other work [14], with body weight being the variable that showed higher correlations with the morphometric measurements. In agreement with the data obtained in *Cichlasoma festae* [14], fish from the rivers had a higher number of significant correlations (*p* < 0.05) than those from the fish farm. These results support the affirmation that the habitat influences the morphology of fish [19]. 

The data standardization following Elliot et al. [20] did not totally remove the fish size effect because all of the transformed variables correlated with the standard length, with the exception of the anal fin length and body width 4. Because of this, Reist standardization was applied [22], showing, in this case, that 10 morphometric variables correlated with the standard length. The adjusted size of the fish, using the transformation of the variables following the regression technique, was successful; this allowed the removal of the variation in size, as well as the minimization of adverse effects [22]. However, this was not in agreement with the present study because the transformation using Elliot et al.’s [18] methodology, which is based on regression, did not work as the effect of the fish size was not removed. Therefore, the Reist methodology [22] was used, which did not work effectively either, though its effectiveness was superior. 

The morphometric measurements were significantly higher (*p* < 0.05) in the cultured fish, specifically in 10 of them (Table 5). Apart from this, the wild fish were significantly greater (*p* < 0.05) in three morphometric measurements (dorsal_2 fin length, anal fin length, and body perimeter 3; Table 5). Conversely, sex significantly influenced (*p* < 0.05) nine variables, with larger values being obtained for males, except for pre_dorsal_2 fin, dorsal_2 fin length, and body width 2 and 3. The interaction of both effects was statistically significant (*p* < 0.05) for dorsal_2 fin ray length and body depth 3.

The discrimination function, obtained with the 17 variables, is shown in Table S4. There were 13 morphometric variables with discrimination power for the production system and sex in *Dormitator latifrons*; they were related to the length, width, and perimeter of the fish, with eight of them being significant (*p* < 0.05).

The percentage of correct adscription to the group was 84%, with higher errors occurring in the wild fish (hit rate of 70.84% and 80.77% in females and males, respectively; data not presented). These errors in the adscription of the wild fish were a consequence of the similarity in the morphostructure between sexes, so four females were better docked with the group of wild males and three males with the group of wild females. Instead, the cultured fish, with 92% correct adscription, showed errors, such as two males that had a morphostructural model closer to the wild male group, a female with the cultured male group, and another one with the wild female group.

**Figure 2:**
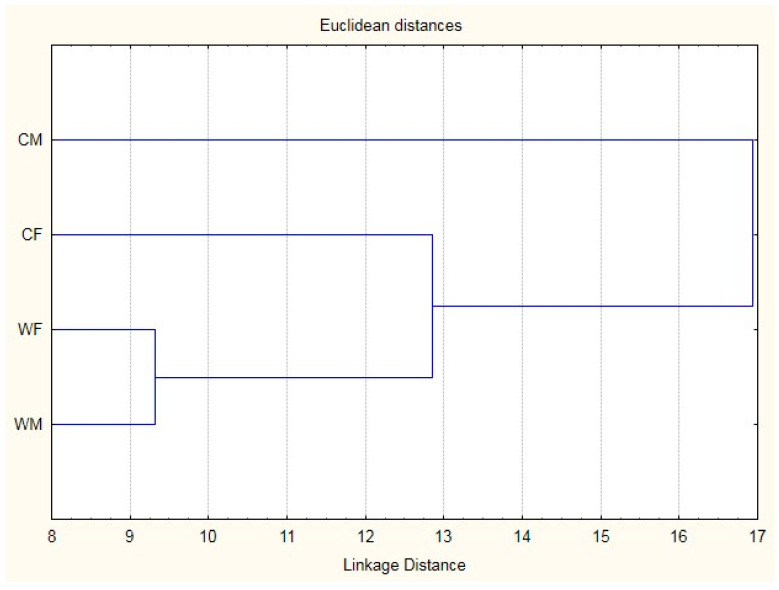
Cluster from Mahalanobis distances in *Dormitator latifrons* for both factors (sex and production system). CM: cultured males; CF: cultured females; WM: wild males; WF: wild females.

The similarities and differences in the morphostructure are shown in Figure 2, which is a graphic representation of Mahalanobis distances. The wild fish are closer, with the cultured males being the farthest group, as these specimens had the most differentiated morphostructure.

The discriminant analysis showed the influence of the production system and sex on the morphostructure of the chame, requiring a small number of variables (17 out of the initial 28 in this study). In several previous works, the effectiveness of this kind of analysis in the differentiation of fish populations has been shown [14,15,19,25]. In a study on *Cichlasoma festae*, it was reported that, on occasion, the causes of the morphological differences among populations are complex; however, in the case of *Cichlasoma festae,* the morphometric differences between the cultured fish and the wild ones could be related to environmental factors [14]. Solomon et al. [25] explained that the fish farming of a particular species for several years can dilute the initial gene pool, driving genetic variation manifested in morphological differences. However, this is not the case for *Dormitator latifrons* because the fingerlings are captured in their natural habitat for later farming, due to reproduction under controlled conditions not having much success [35]. Moreover, Stearns [36] reported that the fish quickly adapt to environmental changes, modifying their physiology and behavior by changing their morphology. Therefore, the differences between both production systems could be explained by the availability of more food for fish on fish farms than in rivers, with the latter depending more on climate conditions.

The development of conservation programs in terms of genetic resources implies morphostructural and productive characterization [8]. Therefore, morphological characterization may be helpful for establishing a conservation program in the future in rivers and on farms. Regarding the conservation of native fish genetic resources, the evaluation of the morphostructural variation shown by a species in different conditions could help to identify the causes responsible for these differences [37]. 

This study has the following limitations: It would be advisable to increase the number of both wild and cultured sampling sites so that the existing variability is increased. Therefore, it is necessary to deepen the production systems and technologies that are being applied.

## 4. Conclusions

The morphological characteristics of *Dormitator latifrons* are influenced by the production system, with larger fish coming from fish farms, where the food availability is greater. Additionally, in this study, this species showed sexual dimorphism, although there were no large significant differences in the morphometric measurements. Meristic counts had a small effect on the production system and sex, with small coefficients of variation. These measures are linked to the species and change very little over time.

The discriminant analysis showed differences between the four defined groups. The cultured males presented the highest differences. The wild animals showed a high heterogeneity, as revealed in the rate of adscription, so it is advised that the morphostructural characteristics of the species that need to be preserved are defined. This study plays an important role in the development of chame breeding and conservation programs.

## Figures and Tables

**Table 1:** Morphometric measurements and meristic counts of *Dormitator latrifrons* used in this study.

Character	Description	Acronyms
Body weight	Total weight including gut and gonads	BW
Total length	Tip of the upper jaw to the caudal end of the caudal fin	TL
Standard length	Tip of the upper jaw to the tail base	SL
Head length	From the front of the upper lip to the posterior end of the opercula membrane	HL
Eye diameter	The greatest bony diameter of the orbit	ED
Preorbital length	Front of the upper lip to cranial eye edge	Pre-OL
Predorsal length	Front of the upper lip to the origin of the dorsal fin	Pre-DL
Prepectoral length	Front of the upper lip to the origin of the pectoral fin	Pre-PcL
Preanal length	Front of the upper lip to the origin of the anal fin	Pre-AL
Dorsal fin length	From base of first dorsal spine to base of last dorsal ray	DFL
Dorsal fin ray length	From base to tip of the fifth dorsal ray	DFRL
Pectoral fin length	From base to tip of the pectoral fin	PcFL
Pelvic fin length	From base to tip of the pelvic fin	PvFL
Anal fin length	From base of first anal spine to base of last anal ray	AFL
Anal fin ray length	From base to tip of the last anal ray	AFRL
Upper jaw length	Straight line measurement between the snout tip and posterior edge of maxilla	UJL
Body depth 1	Body depth at the level of the first ray of the dorsal fin	AC1
Body depth 2	Body depth at the level of the first ray of the anal fin	AC2
Body depth 3	Body depth at the level of the first radius of the caudal fin	AC3
Body perimeter 1	Body perimeter at the level of the first ray of the dorsal fin	P1
Body perimeter 2	Body perimeter at the level of the first radius of the anal fin	P2
Body perimeter 3	Body perimeter at the level of the last ray of the dorsal fin	P3
Body width 1	Straight line measurement from side to side at the level of the base of first dorsal spine	LC1
Body width 2	Straight line measurement from side to side at the level of the base of first anal spine	LC2
Body width 3	Straight line measurement from side to side at the level of the base of last dorsal ray	LC3
Body width 4	Straight line measurement from side to side at the level of the base of last anal ray	LC4
Dorsal fin rays	Number of thorns in the dorsal fin	DFR
Radius dorsal fin	Number of cartilages found in the space between thorns from the dorsal fin	RDF
Pectoral fin rays	Number of thorns in the pectoral fin	PcFR
Radius pectoral fin	Number of cartilages found in the space between thorns in the pectoral fin	RPcF
Pelvic fin rays	Number of thorns in the pelvic fin	PvFR
Radius pelvic fin	Number of cartilages found in the space between thorns in the pelvic fin	RPvF
Anal fin rays	Number of thorns in the anal fin	AFR
Radius anal fin	Number of cartilages found in the space between thorns in the anal fin	RAF
Caudal fin rays	Number of thorns in the caudal fin	CFR
Radius caudal fin	Number of cartilages found in the space between thorns in the caudal fin	RCF

**Table 2:** Descriptive statistics of body measurements (original data) of *Dormitator latrifrons* from hatchery and wild populations for each sex (mean (CV)).

Character ^1^	All (*N* = 300)	Cultured (*N* = 150)	Wild (*N* = 150)	Males (*N* = 153)	Females (*N* = 147)	*p* Value ^2^		
						System Production (A)	Sex (B)	A × B
BW (g)	173.13 (31.00)	209.89 (16.8)	136.38 (31.27)	176.89 (33.26)	169.22 (28.37)	88.57 ***	1.37 ns	0.98 ns
K	2.41 (23.23)	2.48 (30.21)	2.35 (10.76)	2.48 (27.30)	2.34 (17.10)	1.48 ns	1.57 ns	1.99 ns
TL (cm)	24.65 (12.93)	26.46 (9.54)	22.85 (11.99)	24.78 (13.35)	24.53 (12.59)	46.38 ***	0.37 ns	0.01 ns
SL (cm)	19.23 (13.24)	20.65 (9.97)	17.81 (12.22)	19.22 (13.72)	19.24 (12.87)	44.11 ***	0.00 ns	0.05 ns
HL (cm)	6.47 (15.88)	6.93 (12.61)	6.00 (16.02)	6.66 (16.61)	6.26(14.42)	27.05 ***	5.39 *	0.01 ns
ED (cm)	0.88 (18.86)	0.98 (17.14)	0.78 (11.33)	0.87 (22.42)	0.88 (14.56)	52.64 ***	0.01 ns	0.80 ns
Pre-OL (cm)	1.21 (20.23)	1.31 (18.28)	1.11 (18.69)	1.23 (20.78)	1.18 (19.54)	20.68 ***	1.67 ns	0.60 ns
Pre-DL_1 (cm)	7.68 (13.88)	8.25 (10.95)	7.11 (12.75)	7.64 (14.65)	7.72 (13.18)	38.99 ***	0.13 ns	0.67 ns
Pre-DL_2 (cm)	11.53 (12.99)	12.33 (10.49)	10.73 (11.61)	11.42 (13.71)	11.63 (12.3)	39.24 ***	0.48 ns	0.41 ns
Pre-PcL (cm)	6.45 (13.77)	6.88 (11.09)	6.02 (13.19)	6.55 (14.77)	6.34 (12.47)	31.20 ***	2.25 ns	0.07 ns
Pre-AL (cm)	12.95 (14.67)	15.91 (77.75)	11.74 (11.71)	12.89 (15.88)	14.81 (85.69)	5.61 ***	1.09 ns	0.97 ns
DFL_1 (cm)	3.08 (15.26)	3.30 (13.85)	2.85 (12.82)	3.10 (15.73)	3.06 (14.87)	29.03 ***	0.33 ns	0.03 ns
DFL_2 (cm)	0.77 (30.16)	0.78 (33.66)	0.76 (26.34)	0.69 (26.54)	0.85 (29.52)	0.08 ns	12.68 **	0.00 ns
DFRL_1 (cm)	2.22 (18.50)	2.40 (16.97)	2.04 (16.43)	2.27 (20.95)	2.17 (15.17)	22.23 ***	1.79 ns	0.21 ns
DFRL _2 (cm)	2.48 (19.69)	2.72 (19.02)	2.24 (13.94)	2.64 (20.73)	2.31 (15.08)	39.10 ***	19.74 ***	5.39 *
PcFL (cm)	4.51 (15.45)	4.92 (12.07)	4.09 (12.85)	4.55 (16.70	4.46 (14.06)	54.24 ***	1.00 ns	0.01 ns
PvFL (cm)	4.01 (17.86)	4.49 (12.64)	3.53 (13.73)	4.15 (19.04)	3.86 (15.61)	92.34 ***	9.27 **	1.30 ns
AFL (cm)	2.75 (19.09)	2.75 (18.91)	2.76 (19.46)	2.85 (18.26)	2.65 (19.45)	0.00 ns	3.78 ns	0.30 ns
AFRL (cm)	2.03 (35.80)	2.54 (25.35)	1.53 (23.74)	2.11 (34.48)	1.95 (37.20)	96.13 ***	3.09 ns	0.60 ns
UJL (cm)	1.12 (20.18)	1.21 (19.77)	1.03 (16.78)	1.14 (21.01)	1.09 (19.07)	18.41 ***	1.93 ns	0.16 ns
AC1 (cm)	6.45 (17.92)	7.34 (9.74)	5.57 (13.85)	6.45 (18.15)	6.46 (17.85)	139.49 ***	0.03 ns	0.48 ns
AC2 (cm)	5.35 (14.81)	5.81 (10.62)	4.88 (13.8)	5.38 (15.2)	5.31 (14.51)	51.23 ***	0.49 ns	1.33 ns
AC3 (cm)	2.93 (14.14)	3.17 (10.8)	2.69 (12.27)	2.92 (14.51)	2.94 (13.9)	52.72 ***	0.08 ns	1.93 ns
P1 (cm)	14.85 (13.63)	16.22 (7.45)	13.47 (12.76)	14.90 (13.67)	14.79(13.72)	84.33 ***	0.33 ns	0.13 ns
P2 (cm)	12.23 (14.58)	13.49 (8.18)	10.98 (12.87)	12.41 (15.23)	12.05 (13.81)	100.02 ***	2.68 ns	1.05 ns
P3 (cm)	6.79 (11.97)	7.06 (7.29)	6.52 (14.74)	6.83 (9.47)	6.75 (14.24)	11.91 **	0.34 ns	0.08 ns
LC1 (cm)	3.29 (17.75)	3.77 (5.56)	2.81 (15.12)	3.32 (17.61)	3.26 (18.03)	202.69 ***	1.62 ns	0.07 ns
LC2 (cm)	2.89 (13.95)	3.16 (6.30)	2.61 (14.15)	2.83 (13.68)	2.94 (14.05)	84.37 ***	3.14 ns	0.11 ns
LC3 (cm)	2.10 (16.16)	2.32 (10.23)	1.88 (14.83)	2.09 (17.20)	2.11 (15.17)	70.30 ***	0.05 ns	0.00 ns
LC4 (cm)	1.14 (21.33)	1.19 (25.89)	1.11 (14.03)	1.11 (10.78)	1.19 (27.49)	2.40 ns	2.16 ns	3.19 ns

^1^ BW = body weight; K = Fulton’s factor; TL = total length; SL = standard length; HL = head length; ED = eye diameter; Pre-OL = preorbital length; Pre-DL_1 = predorsal_1 length; Pre-DL_2 = predorsal_2 length; Pre-PcL = prepectoral length; Pre-AL = preanal length; DFL_1 = dorsal_1 fin length; DFL_2 = dorsal_2 fin length; DFRL_1 = dorsal_1 fin ray length; DFRL_2 = dorsal_2 fin ray length; PcFL = pectoral fin length; PvFL = pelvic fin length; AFL = anal fin length; AFRL = anal fin ray length; UJL = upper jaw length; AC1 = body depth 1; AC2 = body depth 2; AC3 = body depth 3; P1 = body perimeter 1; P2 = body perimeter 2; P3 = body perimeter 3; LC1 = body width 1; LC2 = body width 2; LC3 = body width 3; LC4 = body width 4. ^2^ * *p* < 0.05; ** *p* < 0.01; *** *p* < 0.001; ns = not significantly different.

**Table 3:** Descriptive statistics of the meristic characters (original data) of *Dormitator latifrons* from hatchery and wild populations for each sex (mean (CV)).

Character ^1^	All (*N* = 300)	Cultured (*N* = 150)	Wild (*N* = 150)	Males (*N* = 153)	Females (*N* = 147)	*p* Value ^2^		
						System Production (A)	Sex (B)	A × B
DFR-1	6.98 (2.02)	6.98 (2.03)	6.98 (2.03)	6.98 (2.01)	6.98 (2.05)	n.a	0.81 ns	1.94 ns
RDF-1	5.98 (2.35)	5.98 (2.36)	5.98 (2.36)	5.98 (2.34)	5.98 (2.39)	n.a	0.81 ns	1.94 ns
DFR-2	9.98 (2.00)	10.02 (1.41)	9.94 (2.41)	9.98 (1.40)	9.98 (2.50)	4.00 *	0.93 ns	5.12 ns
RDF-2	8.98 (2.23)	9.02 (1.57)	8.94 (2.68)	8.98 (1.56)	8.98 (2.77)	4.00 *	0.93 ns	5.12 ns
PcRF	15.04 (3.26)	15.16 (3.62)	14.92 (2.65)	14.94 (3.11)	15.14 (3.30)	6.12 *	4.24 *	10.40 *
RPcF	14.04 (3.50)	14.16 (3.87)	13.92 (2.84)	13.94 (3.34)	14.14 (3.54)	6.12 *	4.24 *	10.40 *
PvRF	5.00 (0)	5 (0)	5 (0)	5 (0)	5 (0)	n.a	n.a	n.a
RPvF	4.00 (0)	4 (0)	4 (0)	4 (0)	4 (0)	n.a	n.a	n.a
AFR	10.84 (4.09)	10.70 (4.72)	10.98 (2.90)	10.78 (3.85)	10.90 (4.29)	10.31 **	1.44 ns	15.56 **
RAF	9.86 (4.56)	9.74 (5.41)	9.98 (3.19)	9.82 (4.42)	9.90 (4.72)	7.59 **	0.61 ns	10.41 *
CFR	15.04 (3.12)	15.06 (3.65)	15.02 (2.51)	15.14 (3.24)	14.94 (2.87)	0.49 ns	4.36 *	5.57 ns
RCF	14.04 (3.35)	14.06 (3.91)	14.02 (2.69)	14.14 (3.47)	13.94 (3.07)	0.49 ns	4.36 *	5.47 ns

^1^ DFR-1 = dorsal_1 fin rays; RDF-1 = radius dorsal_1 fin; DFR-2 = dorsal_2 fin rays; RDF-2 = radius dorsal_2 fin; PcRF = pectoral fin rays; RPcF = radius pectoral fin; PvRF = pelvic fin rays; RPvF = radius pelvic fin; AFR = anal fin rays; RAF = radius anal fin; CFR = caudal fin rays; RCF = radius caudal fin. ^2^ * *p* < 0.05; ** *p* < 0.01; ns = not significantly different; na = not applicable MANOVA.

**Table 4:** Descriptive statistics of the ratio between body measurements and standard length (original data) of *Dormitator latifrons* from hatchery and wild populations for each sex (mean (CV)).

Character ^1^	All (*N* = 300)	Cultured (*N* = 150)	Wild (*N* = 150)	Males (*N* = 153)	Females (*N* = 147)	*p* Value ^2^		
						System Production (A)	Sex (B)	A × B
BW/SL	8.84 (23.81)	10.19 (15.38)	7.48 (22.14)	9.03 (26.05)	8.63 (20.97)	69.70 ***	2.10 ns	1.76 ns
TL/SL	1.28 (1.64)	1.28 (1.80)	1.28 (1.47)	1.29 (1.69)	1.28 (1.35)	0.11 ns	14.54 ***	0.68 ns
HL/SL	0.34 (7.69)	0.34 (7.62)	0.34 (7.83)	0.35 (7.28)	0.33 (6.83)	0.00 ns	17.48 ***	0.05 ns
ED/SL	0.05 (15.62)	0.05 (14.87)	0.04 (15.89)	0.05 (17.39)	0.05 (13.75)	4.21 *	0.15 ns	0.90 ns
Pre-OL/SL	0.06 (17.58)	0.06 (17.08)	0.06 (18.21)	0.06 (16.15)	0.06 (18.98)	0.16 ns	1.60 ns	0.42 ns
Pre-DL_1/SL	0.40 (4.25)	0.40 (4.69)	0.40 (3.79)	0.40 (4.36)	0.40 (4.09)	0.01 ns	1.33 ns	2.74 ns
Pre-DL_2/SL	0.60 (2.91)	0.60 (3.48)	0.60 (2.12)	0.59 (2.84)	0.61 (2.74)	4.28 *	9.33 **	2.22 ns
Pre-PcL/SL	0.34 (6.65)	0.33 (7.09)	0.34 (6.19)	0.34 (5.96)	0.33 (7.03)	0.87 ns	5.59 *	0.12 ns
Pre-AL/SL	0.67 (4.76)	0.69 (4.87)	0.66 (3.78)	0.67 (5.35)	0.68 (4.06)	18.81 ***	1.30 ns	0.64 ns
DFL_1/SL	0.16 (8.48)	0.16 (9.56)	0.16 (7.34)	0.16 (8.06)	0.16 (8.94)	0.13 ns	0.73 ns	0.05 ns
DFL_2/SL	0.04 (29.66)	0.04 (32.63)	0.04 (25.81)	0.04 (26.63)	0.04 (28.82)	5.86 *	13.74 ***	0.00 ns
DFRL_1/SL	0.12 (12.51)	0.12 (14.56)	0.11 (10.1)	0.12 (14.24)	0.11 (9.97)	0.24 ns	2.57 ns	0.70 ns
DFRL_2/SL	0.13(13.43)	0.13 (16.54)	0.13 (8.43)	0.14 (13.00)	0.12 (9.85)	4.75 *	33.16 ***	9.03 **
PcFL/SL	0.23(7.64)	0.24 (7.78)	0.23 (7.12)	0.24 (7.84)	0.23 (7.35)	4.80 *	2.57 ns	0.35 ns
PvFL/SL	0.21 (10.62)	0.22 (10.39)	0.20(8.32)	0.22 (10.69)	0.20 (9.27)	28.65 ***	17.75 ***	3.32 ns
AFL/SL	0.14 (15.35)	0.13 (13.96)	0.15 (12.89)	0.15 (15.00)	0.14 (14.77)	34.28 ***	9.05 **	0.59 ns
AFRL/SL	0.11 (29.76)	0.12 (21.59)	0.09 (28.78)	0.11 (29.45)	0.10 (29.69)	47.93 ***	3.46 ns	0.52 ns
UJL/SL	0.06 (17.46)	0.06 (17.02)	0.06 (18.05)	0.06 (15.69)	0.06 (19.17)	0.00 ns	1.60 ns	0.34 ns
AC1/SL	0.33 (9.51)	0.36 (7.55)	0.31 (5.87)	0.33 (9.17)	0.33 (9.96)	87.57 ***	0.13 ns	0.38 ns
AC2/SL	0.28 (7.30)	0.28 (7.83)	0.27 (6.49)	0.28 (7.17)	0.28 (7.43)	3.25 ns	1.63 ns	2.60 ns
AC3/SL	0.15(5.64)	0.15 (6.21)	0.15 (4.88)	0.15 (5.39)	0.15 (5.93)	2.29 ns	0.23 ns	5.28 *
P1/SL	0.77 (9.72)	0.79 (11.82)	0.76 (5.98)	0.78 (10.3)	0.77 (9.12)	5.09 *	0.59 ns	0.57 ns
P2/SL	0.64 (10.23)	0.66 (11.95)	0.62 (6.34)	0.65 (11.23)	0.63 (8.79)	10.78 **	3.29 ns	2.08 ns
P3/SL	0.36 (11.50)	0.34 (10.37)	0.37 (11.63)	0.36 (9.56)	0.35 (13.33)	8.92 **	0.42 ns	0.29 ns
LC1/SL	0.17 (12.18)	0.18 (9.84)	0.16 (8.99)	0.17 (13.06)	0.17 (11.11)	66.14 ***	1.84 ns	0.00 ns
LC2/SL	0.15 (8.62)	0.15 (9.47)	0.15 (6.79)	0.15 (8.38)	0.15 (8.54)	8.13 **	4.52 *	0.04 ns
LC3/SL	0.11 (10.39)	0.11 (11.08)	0.11 (8.24)	0.11 (11.47)	0.11 (9.25)	10.81 ***	0.05 ns	0.21 ns
LC4/SL	0.06 (23.59)	0.06 (32.8)	0.06 (9.58)	0.06 (12.32)	0.06 (30.38)	2.33 ns	1.75 ns	2.34 ns

^1^ BW = body weight; TL = total length; SL = standard length; HL = head length; ED = eye diameter; Pre-OL = preorbital length; Pre-DL_1 = predorsal_1 length; Pre-DL_2 = predorsal_2 length; Pre-PcL = prepectoral length; Pre-AL = preanal length; DFL_1 = dorsal_1 fin length; DFL_2 = dorsal_2 fin length; DFRL_1 = dorsal_1 fin ray length; DFRL_2 = dorsal_2 fin ray length; PcFL = pectoral fin length; PvFL = pelvic fin length; AFL = anal fin length; AFRL = anal fin ray length; UJL = upper jaw length; AC1 = body depth 1; AC2 = body depth 2; AC3 = body depth 3; P1 = body perimeter 1; P2 = body perimeter 2; P3 = body perimeter 3; LC1 = body width 1; LC2 = body width 2; LC3 = body width 3; LC4 = body width 4. ^2^ * *p* < 0.05; ** *p* < 0.01; *** *p* < 0.001; ns = not significantly different.

**Table 5:** Descriptive statistics of body measurements (adjusted data) of *Dormitator latifrons* from hatchery and wild populations for each sex (mean (CV)).

Character ^1^	All (*N* = 300)	Cultured (*N* = 150)	Wild (*N* = 150)	Males (*N* = 153)	Females (*N* = 147)	*p* Value ^2^		
						System Production (A)	Sex (B)	A × B
BW (g)	8.84 (23.81)	10.19 (15.38)	7.48 (22.14)	9.03 (26.05)	8.63 (20.97)	69.70 ***	2.10 ns	1.76 ns
K	0.13 (32.16)	0.12 (41.73)	0.13 (20.41)	0.13 (36.33)	0.13 (26.34)	1.55 ns	1.05 ns	1.66 ns
TL (cm)	1.28 (1.64)	1.28 (1.80)	1.28 (1.47)	1.29 (1.69)	1.28 (1.35)	0.11 ns	14.54 ***	0.68 ns
HL (cm)	0.34 (7.69)	0.34 (7.62)	0.34 (7.83)	0.35 (7.28)	0.33 (6.83)	0.00 ns	17.48 ***	0.05 ns
ED (cm)	0.05 (15.62)	0.05 (14.87)	0.04 (15.89)	0.05 (17.39)	0.05 (13.75)	4.21 *	0.15 ns	0.90 ns
Pre-OL (cm)	0.06 (17.58)	0.06 (17.08)	0.06 (18.21)	0.06 (16.15)	0.06 (18.98)	0.16 ns	1.60 ns	0.42 ns
Pre-DL_1 (cm)	0.40 (4.25)	0.40 (4.69)	0.40 (3.79)	0.40 (4.36)	0.40 (4.09)	0.01 ns	1.33 ns	2.74 ns
Pre-DL_2 (cm)	0.60 (2.91)	0.60 (3.48)	0.60 (2.12)	0.59 (2.84)	0.61 (2.74)	4.28 *	9.33 **	2.22 ns
Pre-PcL (cm)	0.34 (6.65)	0.33 (7.09)	0.34 (6.19)	0.34 (5.96)	0.33 (7.03)	0.87 ns	5.59 *	0.12 ns
Pre-AL (cm)	0.67 (4.76)	0.69 (4.87)	0.66 (3.78)	0.67 (5.35)	0.68 (4.06)	18.81 ***	1.30 ns	0.64 ns
DFL_1 (cm)	0.16 (8.48)	0.16 (9.56)	0.16 (7.34)	0.16 (8.06)	0.16 (8.94)	0.13 ns	0.73 ns	0.05 ns
DFL_2 (cm)	0.04 (29.66)	0.04 (32.63)	0.04 (25.81)	0.04 (26.63)	0.04 (28.82)	5.86 *	13.74 ***	0.00 ns
DFRL_1 (cm)	0.12 (12.51)	0.12 (14.56)	0.11 (10.1)	0.12 (14.24)	0.11 (9.97)	0.24 ns	2.57 ns	0.70 ns
DFRL _2 (cm)	0.13 (13.43)	0.13 (16.54)	0.13 (8.43)	0.14 (13.00)	0.12 (9.85)	4.75 *	33.16 ***	9.03**
PcFL (cm)	0.23 (7.64)	0.24 (7.78)	0.23 (7.12)	0.24 (7.84)	0.23 (7.35)	4.80 *	2.57 ns	0.35 ns
PvFL (cm)	0.21 (10.62)	0.22 (10.39)	0.20 (8.32)	0.22 (10.69)	0.20 (9.27)	28.65 ***	17.75 ***	3.32 ns
AFL (cm)	0.14 (15.35)	0.13 (13.96)	0.15 (12.89)	0.15 (15.00)	0.14 (14.77)	34.28 ***	9.05 **	0.59 ns
AFRL (cm)	0.11 (29.76)	0.12 (21.59)	0.09 (28.78)	0.11 (29.45)	0.10 (29.69)	47.93 ***	3.46 ns	0.52 ns
UJL (cm)	0.06 (17.46)	0.06 (17.02)	0.06 (18.05)	0.06 (15.69)	0.06 (19.17)	0.00 ns	1.60 ns	0.34 ns
AC1 (cm)	0.33 (9.51)	0.36 (7.55)	0.31 (5.87)	0.33 (9.17)	0.33 (9.96)	87.57 ***	0.13 ns	0.38 ns
AC2 (cm)	0.28 (7.30)	0.28 (7.83)	0.27 (6.49)	0.28 (7.17)	0.28 (7.43)	3.25 ns	1.63 ns	2.60 ns
AC3 (cm)	0.15 (5.64)	0.15 (6.21)	0.15 (4.88)	0.15 (5.39)	0.15 (5.93)	2.29 ns	0.23 ns	5.28*
P1 (cm)	0.77 (9.72)	0.79 (11.82)	0.76 (5.98)	0.78 (10.3)	0.77 (9.12)	5.09 *	0.59 ns	0.57 ns
P2 (cm)	0.64 (10.23)	0.66 (11.95)	0.62 (6.34)	0.65 (11.23)	0.63 (8.79)	10.78 **	3.29 ns	2.08 ns
P3 (cm)	0.36 (11.50)	0.34 (10.37)	0.37 (11.63)	0.36 (9.56)	0.35 (13.33)	8.92 **	0.42 ns	0.29 ns
LC1 (cm)	0.17 (12.18)	0.18 (9.84)	0.16 (8.99)	0.17 (13.06)	0.17 (11.11)	66.14 ***	1.84 ns	0.00 ns
LC2 (cm)	0.15 (8.62)	0.15 (9.47)	0.15 (6.79)	0.15 (8.38)	0.15 (8.54)	8.13 **	4.52 *	0.04 ns
LC3 (cm)	0.11 (10.39)	0.11 (11.08)	0.11 (8.24)	0.11 (11.47)	0.11 (9.25)	10.81 **	0.05 ns	0.21 ns
LC4 (cm)	0.06 (23.59)	0.06 (32.8)	0.06 (9.58)	0.06 (12.32)	0.06 (30.38)	2.33 ns	1.75 ns	2.34 ns

^1^ BW = body weight; K = Fulton’s factor; TL = total length; HL = head length; ED = eye diameter; Pre-OL = preorbital length; Pre-DL_1 = predorsal_1 length; Pre-DL_2 = predorsal_2 length; Pre-PcL = prepectoral length; Pre-AL = preanal length; DFL_1 = dorsal_1 fin length; DFL_2 = dorsal_2 fin length; DFRL_1 = dorsal_1 fin ray length; DFRL_2 = dorsal_2 fin ray length; PcFL = pectoral fin length; PvFL = pelvic fin length; AFL = anal fin length; AFRL = anal fin ray length; UJL = upper jaw length; AC1 = body depth 1; AC2 = body depth 2; AC3 = body depth 3; P1 = body perimeter 1; P2 = body perimeter 2; P3 = body perimeter 3; LC1 = body width 1; LC2 = body width 2; LC3 = body width 3; LC4 = body width 4. ^2^ * *p* < 0.05; ** *p* < 0.01; *** *p* < 0.001; ns = not significantly different.

## Supplementary Materials

The following are available online at https://www.mdpi.com/2076-2615/10/10/1805/s1, Table S1: Concentrate food for shrimp produced by Agripac S.A; Table S2: Pearson correlation coefficients between morphometric measures of *Dormitator latifrons* from cultured populations in both sexes (males above diagonal/females upper diagonal); Table S3: Pearson correlation coefficients between morphometric measures of *Dormitator latifrons* from wild populations in both sexes (males above diagonal/females upper diagonal); Table S4: Discrimination function of the morphometric variables (fitted data) for both sexes, as well as for wild and cultured *Dormitator latifrons*; Figure S1: Geographic distribution of *Dormitator latifrons*; Figure S2: Sites of capture of *Dormitator latifrons*: wild (left) and cultured (right); Figure S3: Cultured pool of *Dormitator latifrons*.
