# Supplementary material for: Morphometric and Meristic Characterization of Native Chame Fish (Dormitator latifrons) in Ecuador Using Multivariate Analysis
Source: Animals (Basel). 2020 Oct 4;10(10):1805. doi: 10.3390/ani10101805 (PMC7599706; doi:10.3390/ani10101805)
Supplement: Supplementary file 1 [file animals-10-01805-s001.pdf]

Table S1. Concentrate food for shrimp produced by Agripac S.A.

Percentage	Start<br>Young 1	Growth 1<br>Young 2	Growth 2<br>Adult 1	Fattening<br>Adult 2
Crude protein (min.)	35.0%	33.0%	28.0%	22.0%
Crude fat (min.)	6.5%	6.5%	6.0%	5.0%
Crude fiber (max.)	4.0%	4.0%	5.0%	5.0%
Ash (max.)	12.0%	12.5%	11.0%	11.0%
Humidity (max.)	11.0%	12.0%	11.0%	11.0%

Table S2. Pearson correlation coefficients between morphometric measures of *Dormitator latifrons* from cultured populations in both sexes (males above diagonal/females upper diagonal).

Character[1]	TL	SL	HL	Pre-DL_1	Pre-DL_2	DFL_1	DFRL_1	DFL_2	DFRL_2	AFL	AFRL	PvFL	PcFRL	ED	UJL	Pre-OL	Pre-PcL	Pre-AL	P1	P2	P3	LC1	LC2	LC3	LC4	AC1	AC2	AC3
TL		***	***	***	***	***	**	*	**	***	**	***	***	**	***	**	***	***	ns	ns	*	ns	**	*	ns	***	***	***
SL	***		***	***	***	***	*	*	**	***	**	***	***	**	***	*	***	***	ns	ns	*	*	**	*	*	***	***	***
HL	***	***		***	***	**	**	**	***	**	**	***	***	***	**	***	***	***	ns	ns	ns	*	ns	ns	ns	***	***	***
Pre-DL_1	***	***	***		***	***	***	**	***	**	**	***	***	***	**	**	***	***	ns	ns	*	*	*	ns	ns	***	***	***
Pre-DL_2	***	***	***	***		***	**	**	**	***	**	***	***	**	**	**	***	***	ns	ns	*	*	**	*	*	***	***	***
DFL_1	**	**	*	**	***		ns	ns	ns	***	*	***	**	*	**	ns	**	***	**	*	***	ns	***	***	**	***	***	***
DFRL_1	**	**	*	**	**	ns		ns	**	ns	ns	*	***	***	ns	*	**	**	ns	ns	ns	ns	ns	ns	ns	**	**	**
DFL_2	ns	ns	ns	ns	ns	**	ns		ns	ns	ns	ns	ns	ns	ns	**	*	*	ns	ns	ns	ns	ns	ns	ns	ns	ns	ns
DFRL_2	***	**	*	**	**	ns	***	ns		**	*	***	**	*	ns	**	**	*	ns	ns	ns	ns	ns	ns	ns	ns	*	**
AFL	***	***	**	***	***	**	ns	ns	ns		*	***	**	ns	**	ns	**	*	ns	ns	**	ns	*	ns	*	**	***	***
AFRL	**	**	*	ns	*	*	*	ns	**	ns		**	**	ns	ns	*	**	**	ns	ns	ns	ns	ns	ns	ns	ns	ns	*
PvFL	**	*	**	**	*	ns	**	ns	***	ns	*		***	**	ns	*	***	***	ns	ns	**	ns	**	*	*	**	***	***
PcFRL	***	***	**	***	**	ns	***	ns	**	*	*	**		***	**	*	***	***	ns	ns	ns	ns	*	*	*	***	***	***
ED	ns	ns	*	*	*	ns	ns	ns	*	ns	ns	*	ns		*	ns	**	**	ns	ns	ns	ns	ns	ns	*	**	**	**
UJL	ns	ns	ns	ns	ns	**	*	**	ns	ns	**	ns	ns	ns	ns	ns	**	**	ns	ns	ns	ns	*	ns	**	*	**	***
Pre-OL	ns	ns	*	ns	ns	ns	ns	ns	ns	ns	ns	ns	ns	ns	ns		***	*	ns	ns	ns	ns	ns	ns	ns	*	ns	*
Pre-PcL	***	**	***	***	***	ns	ns	ns	ns	ns	ns	ns	ns	*	ns	*		***	ns	ns	ns	ns	*	ns	ns	***	***	***
Pre-AL	ns	ns	ns	ns	ns	ns	ns	ns	ns	ns	ns	ns	ns	ns	ns	ns	ns		ns	ns	ns	*	*	*	ns	***	**	***
P1	ns	ns	ns	ns	ns	ns	*	ns	ns	ns	ns	ns	ns	ns	ns	ns	ns	ns		***	***	ns	**	**	*	**	**	ns
P2	ns	ns	ns	ns	ns	ns	ns	ns	ns	ns	ns	ns	ns	ns	ns	ns	ns	ns	**		***	ns	**	*	ns	**	**	*
P3	ns	ns	ns	ns	ns	ns	ns	ns	ns	ns	ns	ns	ns	ns	ns	ns	ns	ns	*	*		ns	**	ns	**	*	**	**
LC1	*	*	ns	ns	ns	ns	ns	ns	ns	ns	ns	ns	ns	ns	ns	ns	*	*	ns	ns	ns		**	*	ns	ns	ns	ns
LC2	ns	ns	ns	ns	ns	ns	ns	ns	ns	ns	ns	ns	ns	ns	ns	ns	ns	ns	ns	ns	ns	**		**	*	**	**	**

LC3	ns	ns	ns	ns	*	ns	ns	ns	ns	ns	ns	ns	ns	ns	ns	ns	ns	ns	ns	ns	**	*	**		ns	**	**	ns
LC4	ns	ns	ns	ns	ns	ns	ns	ns	ns	ns	ns	ns	ns	ns	ns	ns	ns	ns	ns	ns	ns	ns	ns	ns		*	*	**
AC1	**	**	**	**	***	**	ns	ns	ns	ns	*	ns	*	ns	ns	ns	**	ns	*	**	ns	*	ns	ns	ns	*	**	***
AC2	***	***	**	**	**	*	ns	ns	ns	***	ns	ns	*	ns	ns	ns	**	ns	ns	**	ns	ns	ns	ns	ns		***	***
AC3	***	***	***	***	***	**	**	ns	**	**	**	**	***	ns	*	ns	**	ns	ns	ns	ns	ns	ns	ns	ns	***	***	

[1] BW = Body weight; TL = Total length; SL = Standard length; HL = Head length; ED = Eye diameter; Pre-OL = Pre-orbital length; Pre-DL_1 = Pre-dorsal_1 length; Pre-DL_2 = Pre-dorsal_2 length; Pre-PcL = Pre-pectoral length; Pre-AL = Pre-anal length; DFL_1 = Dorsal_1 fin length; DFL_2 = Dorsal_2 fin length; DFRL_1 = Dorsa_1l fin ray length; DFRL_2 = Dorsal_2 fin ray length; PcFL = Pectoral fin length; PvFL = Pelvic fin length; AFL = Anal fin length; AFRL = Anal fin ray length; UJL = Upper jaw length; AC1 = Body depth 1; AC2 = Body depth 2; AC3 = Body depth 3; P1 = Body perimeter 1; P2 = Body perimeter 2; P3 = Body perimeter 3; LC1 = Body width 1; LC2 = Body width 2; LC3 = Body width 3; LC4 = Body width 4. * $p < 0.05$; ** $p < 0.01$; *** $p < 0.001$; ns = not significantly different.

Table S3. Pearson correlation coefficients between morphometric measures of *Dormitator latifrons* from wild populations in both sexes (males above diagonal/females upper diagonal).

Character [1]	TL	SL	HL	Pre-DL_1	Pre-DL_2	DFL_1	DFRL_1	DFL_2	DFRL_2	AFL	AFRL	PvFL	PcFRL	ED	UJL	Pre-OL	Pre-PcL	Pre-AL	P1	P2	P3	LC1	LC2	LC3	LC4	AC1	AC2	AC3
TL		***	***	***	***	***	***	ns	***	***	ns	***	***	ns	*	**	***	***	***	***	***	***	***	***	***	***	***	***
SL	***		***	***	***	***	***	ns	***	***	ns	***	***	ns	*	**	***	***	***	***	***	***	***	***	***	***	***	***
HL	***	***		***	***	***	***	ns	***	***	ns	***	***	ns	*	***	***	***	***	***	***	***	***	***	***	***	***	***
Pre-DL_1	***	***	***		***	***	***	ns	***	***	ns	***	***	ns	*	***	***	***	***	***	***	***	***	***	***	***	***	***
Pre-DL_2	***	***	***	***		***	***	ns	***	***	ns	***	***	ns	*	**	***	***	***	***	***	***	***	***	***	***	***	***
DFL_1	***	***	***	***	***		***	ns	***	***	ns	**	***	ns	ns	*	***	***	***	***	***	***	***	***	***	***	***	***
DFRL_1	***	***	***	***	***	**		ns	***	**	ns	***	***	ns	ns	*	***	***	***	***	***	***	***	***	**	***	***	***
DFL_2	*	*	ns	ns	*	ns	*		ns	ns	ns	ns	ns	ns	ns	ns	ns	ns	ns	ns	ns	ns	ns	ns	ns	ns	ns	ns
DFRL_2	***	***	***	***	***	***	***	ns		**	ns	***	***	ns	ns	*	***	***	***	***	***	***	***	***	**	***	***	***
AFL	***	***	***	***	***	***	***	*	***		ns	***	***	ns	ns	ns	***	***	***	***	**	*	***	***	**	***	***	***
AFRL	ns	ns	ns	ns	ns	ns	ns	ns	ns	ns		ns	ns	ns	ns	ns	ns	ns	ns	ns	ns	ns	ns	ns	ns	ns	ns	ns
PvFL	***	***	***	***	***	***	***	ns	***	**	ns		***	ns	ns	**	***	***	***	***	**	***	***	***	***	***	***	***
PcFRL	***	***	**	***	***	***	***	*	***	*	ns	***		ns	ns	**	***	***	***	***	***	***	***	***	***	***	***	***
ED	ns	ns	ns	ns	ns	ns	ns	ns	ns	ns	ns	ns	ns		ns	ns	ns	ns	ns	ns	ns	ns	ns	ns	*	ns	ns	ns
UJL	ns	ns	ns	ns	ns	ns	ns	ns	ns	*	ns	ns	ns	ns		ns	*	*	*	*	ns	ns	*	*	*	*	*	*
Pre-OL	ns	ns	**	ns	ns	ns	*	ns	*	ns	ns	ns	ns	ns	ns		***	***	**	**	***	***	**	**	*	**	*	**
Pre-PcL	***	***	***	***	***	***	***	ns	***	***	ns	***	**	ns	ns	**		***	***	***	***	***	***	***	***	***	***	***
Pre-AL	***	***	***	***	***	***	***	*	***	***	ns	***	***	ns	ns	ns	***		***	***	***	***	***	***	***	***	***	***
P1	***	***	***	***	***	***	***	*	***	**	ns	***	***	ns	ns	ns	***	***		***	***	***	***	***	***	***	***	***
P2	***	***	***	***	***	***	***	*	***	**	ns	***	***	ns	ns	ns	**	***	***		***	***	***	***	***	***	***	***
P3	**	**	ns	*	**	**	**	ns	**	*	ns	***	**	ns	ns	ns	ns	*	***	***		***	***	***	***	***	***	***
LC1	***	***	ns	***	***	***	***	**	***	**	ns	***	***	ns	ns	ns	***	***	***	***	**		***	***	***	***	***	***
LC2	***	***	ns	***	***	***	**	*	***	**	ns	***	***	ns	ns	ns	***	***	***	***	*	***		***	***	***	***	***
LC3	***	***	ns	***	***	***	**	*	***	**	ns	***	***	ns	ns	ns	***	***	***	***	*	***	***		***	***	***	***

LC4	***	***	ns	***	***	**	*	**	**	*	ns	***	***	ns	ns	ns	**	***	***	***	ns	***	***	***		***	***	***
AC1	***	***	ns	***	***	***	***	*	***	***	ns	***	***	ns	ns	ns	***	***	***	***	*	***	***	***	***		***	***
AC2	***	***	ns	***	***	***	***	*	***	**	ns	***	***	ns	ns	ns	***	***	***	***	**	***	***	***	***	***		***
AC3	***	***	ns	***	***	***	***	**	***	**	ns	***	***	ns	ns	ns	***	***	***	***	**	***	***	***	***	***	***	

[1] BW = Body weight; TL = Total length; SL = Standard length; HL = Head length; ED = Eye diameter; Pre-OL = Pre-orbital length; Pre-DL_1 = Pre-dorsal_1 length; Pre-DL_2 = Pre-dorsal_2 length; Pre-PcL = Pre-pectoral length; Pre-AL = Pre-anal length; DFL_1 = Dorsal_1 fin length; DFL_2 = Dorsal_2 fin length; DFRL_1 = Dorsa_1l fin ray length; DFRL_2 = Dorsal_2 fin ray length; PcFL = Pectoral fin length; PvFL = Pelvic fin length; AFL = Anal fin length; AFRL = Anal fin ray length; UJL = Upper jaw length; AC1 = Body depth 1; AC2 = Body depth 2; AC3 = Body depth 3; P1 = Body perimeter 1; P2 = Body perimeter 2; P3 = Body perimeter 3; LC1 = Body width 1; LC2 = Body width 2; LC3 = Body width 3; LC4 = Body width 4. * $p < 0.05$; ** $p < 0.01$; *** $p < 0.001$; ns = not significantly different.

Table S4. Discrimination function of the morphometric variables (fitted data) for both sexes as well as for wild, and cultured *Dormitator latifrons*.

Character [1]	Wilks'-Lambda	Partial-Lambda	F-remove-(3.83)	*p*-level	Toler.	1-Toler.-(R-Sqr.)
LC1	0.15	0.76	8.53	0.000	0.42	0.58
DFRL_2	0.13	0.86	4.57	0.005	0.63	0.37
AFRL	0.12	0.92	2.36	0.077	0.72	0.28
AFL	0.13	0.84	5.40	0.002	0.56	0.44
HL	0.13	0.88	3.64	0.016	0.34	0.66
Pre-DL_1	0.15	0.77	8.45	0.000	0.33	0.67
PvFL	0.13	0.85	4.71	0.004	0.57	0.43
LC2	0.13	0.84	5.28	0.002	0.37	0.63
AC3	0.12	0.91	2.87	0.041	0.40	0.60
AC2	0.12	0.91	2.69	0.051	0.45	0.55
Pre-PcL	0.12	0.93	2.10	0.106	0.37	0.63
PcFL	0.12	0.94	1.68	0.177	0.59	0.41
UJL	0.12	0.95	1.32	0.273	0.80	0.20

[1] HL = Head length; Pre-DL_1 = Pre-dorsal_1 length; Pre-PcL = Pre-pectoral length; DFRL_2 = Dorsal_2 fin ray length; PcFL = Pectoral fin length; PvFL = Pelvic fin length; AFL = Anal fin length; AFRL = Anal fin ray length; UJL = Upper jaw length; AC2 = Body depth 2; AC3 = Body depth 3; LC1 = Body width 1; LC2 = Body width 2

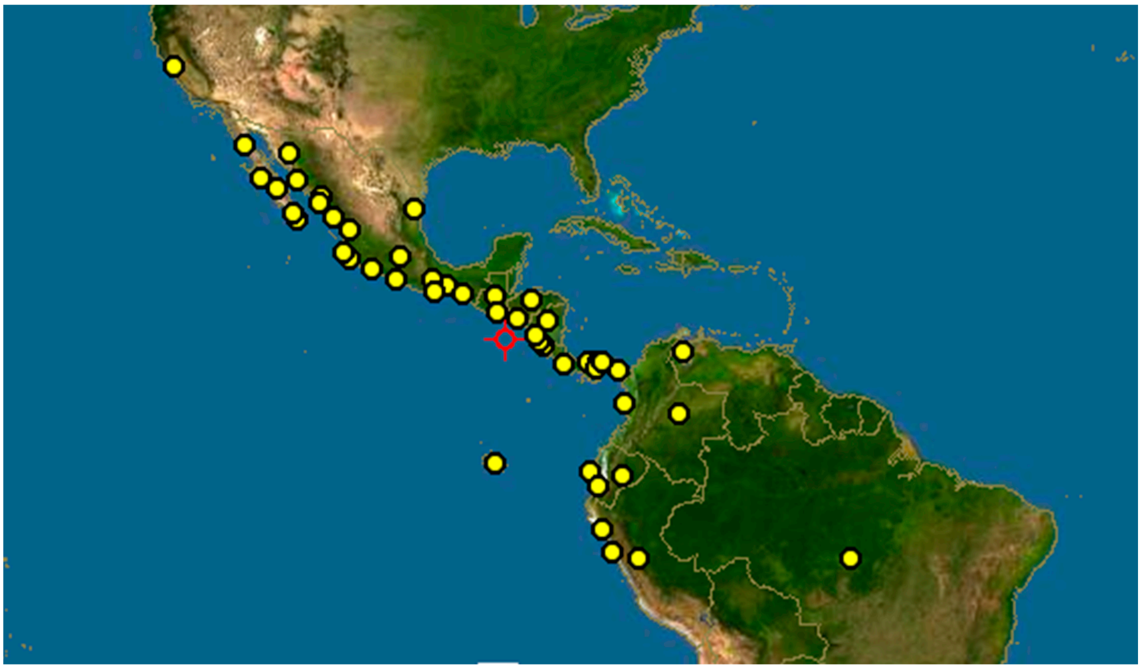

Figure S1. Geographic distribution of *Dormitator latifrons*.

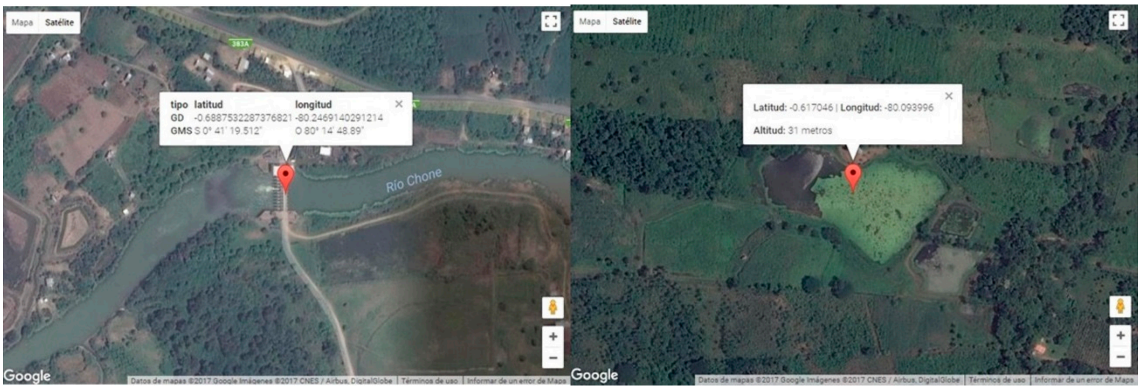

Figure S2. Sites of capture of *Dormitator latifrons*: wild (left) y cultured (right).

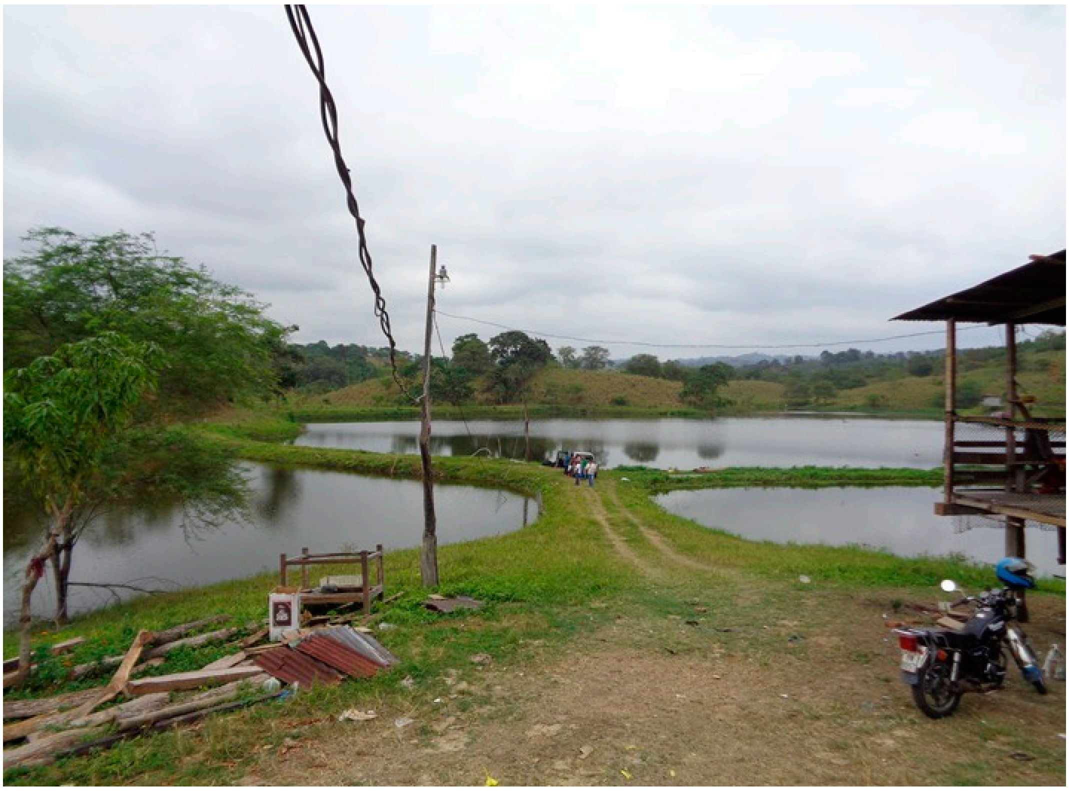

Figure S3. Cultured pool of *Dormitator latifrons*.
